# The effect of Anesthesia Information Management Systems (AIMS) on perioperative epidural documentation: A retrospective cohort study

**DOI:** 10.1177/20552076261492824

**Published:** 2026-09-24

**Authors:** Dulmi Nawarathna, John Shaker, Thomas P. Wall

**Affiliations:** 1 Department of Anaesthesiology & Perioperative Medicine, 8881 Mater Misericordiae University Hospital, Dublin, Ireland; 2 School of Medicine, 91092 University College Dublin, Dublin, Ireland

**Keywords:** Anesthesia, Epidural documentation, Anesthesia information management system, Electronic health record

## Abstract

**Objectives:**

Perioperative epidural documentation is an essential record of a procedure which has significant clinical and medico-legal implications. Little is known about the impact of the introduction of electronic Anesthesia Information Management Systems (AIMS) on the quality of epidural documentation compared to handwritten records. This study identifies elements of epidural documentation and, using a novel scoring system (Documentation Completion Score, DCS) quantifies the difference in documentation completeness between handwritten and AIMS records.

**Methods:**

In a single-centre retrospective cohort study, 100 handwritten anesthesia records involving epidural placement were randomly selected from historic paper charts and a further 100 electronic records were randomly chosen from the AIMS database. All records retrieved were assessed for 15 pre-identified documentation components. DCS was calculated for each record and the groups were compared using inferential statistical tests.

**Results:**

Of 15 documentation components, 11 were more consistently entered in AIMS compared to handwritten records. DCS was significantly higher in the AIMS group (13.48 ± 1.01, mean ± standard deviation) versus the handwritten group (9.91 ± 2.93), *p* < 0.001. Notably, the one element which was more consistently documented in handwritten records (‘LA test dose documented’) did not have a corresponding specific interface element in AIMS.

**Conclusion:**

In our institution, completion of epidural documentation components is more consistent in AIMS compared to handwritten records, although local factors (e.g. user training) may limit the generalisability of this finding. Not including all desired elements on a single page can lead to user failure to document them in other areas of the AIMS chart. Such user behaviour should be considered when configuring AIMS interfaces.

## Introduction

Anesthesia documentation is a fundamental requirement of clinical practice and is vital for maintaining patient safety and continuity of high-quality care, as well as medicolegal protection, reimbursement and research.^1^ Ever since 1894 when Codman and Cushing first used handwritten records to document the physiologic status of patients undergoing anesthesia, paper has remained as the most common method of recording anesthetic management and patient physiological status worldwide.^2^ However, as affordable information technology has proliferated in recent decades, use of software packages to provide an electronic means of recording the perioperative period has become increasingly widespread. Such applications, known as Anesthesia Information Management Systems (AIMS), have been widely adopted by many anesthesiology groups due to perceived positive benefits for providers, patients, and hospitals.^3,4^ Maintaining quality real-time documentation of a patient’s peri-operative course presents a challenge not commonly encountered in other specialties, particularly in complex, highly dynamic cases such as transplants and emergency cardiac or obstetric procedures. A well-configured AIMS can help to reduce the documentation workload on the busy anaesthesiologist while enhancing the accuracy of the recorded data.

Epidural analgesia is a widely established method of pain management for major thoracic and abdominal surgeries as well as labor anesthesia and analgesia.^5,6^ Although new regional anesthesia techniques have evolved in recent years, epidural analgesia is still considered to be the gold standard of pain management for open thoraco-abdominal surgeries.^7^ However, epidural placement is associated with a risk of serious complications such as epidural abscess, meningitis, nerve injury, epidural hematoma, and paralysis – thankfully permanent injury is rare, with the 3^rd^ National Anesthesia Project (NAP3) study in the UK estimating an incidence of between 2.0 and 4.2 per 100,000 neuraxial blocks (i.e. spinal and epidural procedures).^8^ These complications can be devastating for the patient and unsurprisingly form a significant proportion of malpractice claims against anesthesiologists.^9^ Regional and neuraxial anesthesia-related claims composed 54% of cases in one closed-claims study from Switzerland and 24.5% in study from Italy (where anesthesiologists may face not only civil but also criminal charges resulting from adverse events).^10,11^

Clear and comprehensive documentation of epidural placement details, whether on paper or in AIMS, is crucial for safely managing epidural catheters in the postoperative period and for preventing some of the above-mentioned serious complications. Additionally, this documentation serves as a vital piece of evidence supporting appropriate anesthetic management in the event of legal claims. One UK-based expert witness urges that neuraxial procedures “…should be documented meticulously, including the number of attempts, any symptoms elicited, actions taken in light of these …significant negatives should also be recorded, for example, ‘No paraesthesia, no complications’.^12^ What exactly comprises ‘meticulous documentation’ has been less clear, and has historically been largely left to the judgment of the individual practitioner, with recommendations or proforma documents provided by some centers. In recent years, more comprehensive guidance has been published in attempts to develop a standard for anesthesia documentation. An international regional anesthesia expert panel produced a consensus recommendation for effective documentation in regional anesthesia in 2022, with an extensive set of documentation items including 11 related specifically to neuraxial procedures.^13^ In the UK, the Professional Record Standards Body (PRSB), anticipating a widespread move to electronic health records, has very recently published a standard for anesthesia documentation including detailed guidance for neuraxial procedures (involving 16 items).^14^

Understandably, given the historical lack of standards-based guidance, in our institution we noted wide variation in how epidural placements were previously documented in handwritten records. Our paper records provided a generic blank area for documenting procedures, such as airway management, vascular access and neuraxial procedures including epidural placement. Epidural procedural notes could vary from something as basic as ‘*Thoracic epidural - uneventful insertion*’ to highly detailed descriptions of aseptic measures, levels attempted, difficulties encountered, test dose responses etc. Following the introduction of an AIMS system in 2017, anesthesiologists in our center are now presented with a single application page (the ‘Central blocks’ tab, see Figure 1) on which to document neuraxial blocks, largely through the use of single item drop-down boxes, checkboxes or simple numeric entry fields. This study aims to determine whether AIMS introduction improves the quality of documentation of epidural placement compared to previous handwritten records.

**Figure 1.**
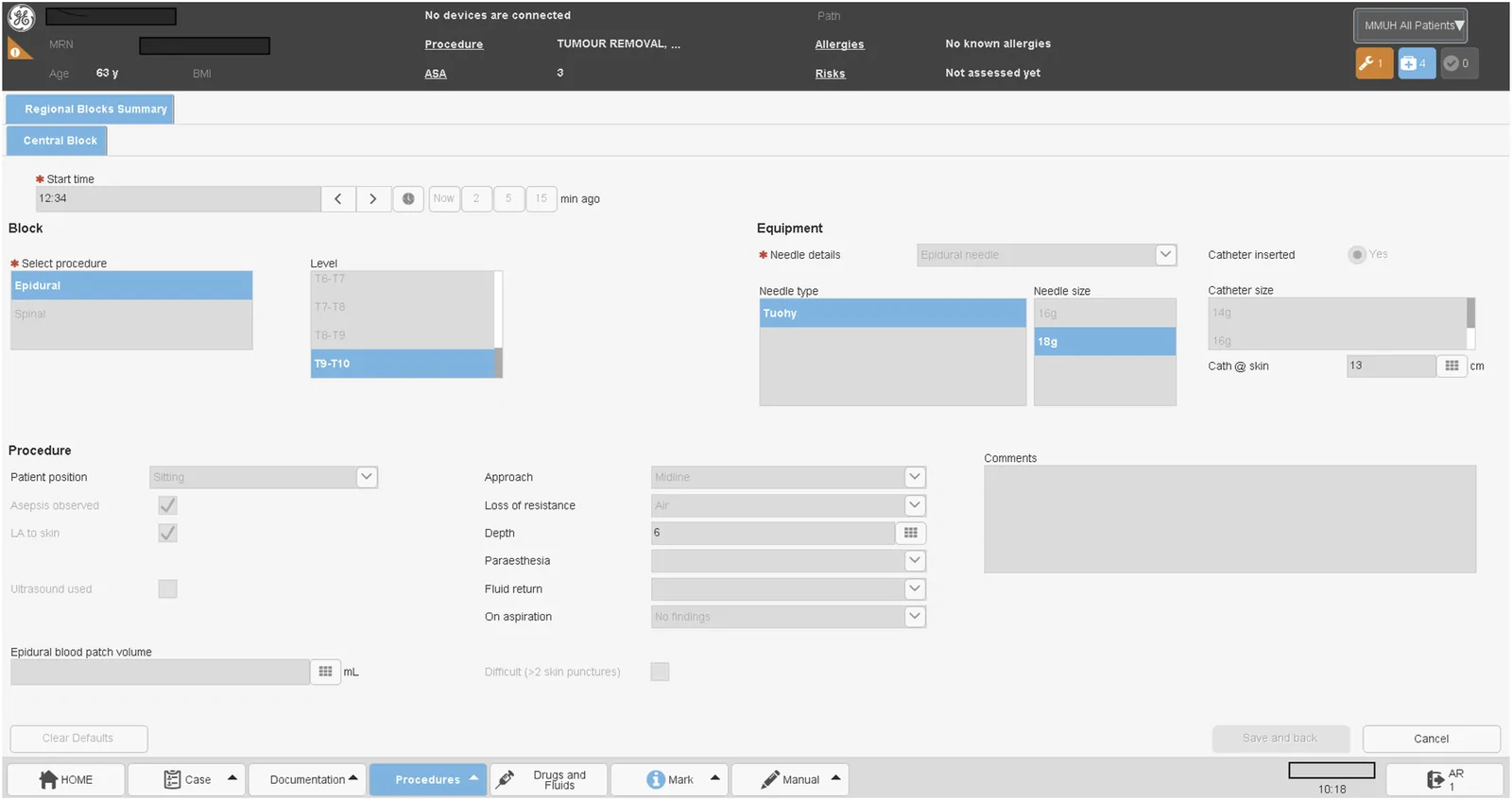
Sample ‘Central blocks’ tab from Centricity AIMS application, configured locally.

## Methods

This was a single centre retrospective cohort study and was reported according to the Strengthening the Reporting of Observational Studies in Epidemiology (STROBE) statement.^15^ A completed STROBE checklist is provided in Supplementary Table 1. Retrospective data concerning the documentation of perioperative epidural catheter placement was collected from both paper charts and Centricity AIMS (GE Healthcare, Chicago, IL, USA). For the handwritten anesthesia records, charts of patients who received perioperative epidurals in the 24 months from January 2015 to December 2016 were selected at random. A random date generation system was used to select a date, all patients receiving epidurals on that date were identified from central operating room registers, and a patient was randomly selected from these (randomization functions were provided by https://www.random.org/). For AIMS records, the Centricity database was queried using Structured Query Language (SQL) commands to retrieve a list of all epidural placements performed within 24 months (July 2017 to July 2019). AIMS records were selected at random from this list using a similar random number generation system.

Using the guidance of the consensus guidelines by Ahmed et al.,^13^ 15 documentation components specific to epidural placement were selected (Table 1). Note that components typically specified elsewhere in the AIMS or handwritten record were not included – this included patient name, date of birth, monitors applied, baseline vitals, intravenous access etc. In the handwritten records, the total number of words used to describe epidural insertion was assessed. Both handwritten and AIMS records were analyzed for the presence or absence of the 15 selected components. Two reviewers (JS, TPW) reviewed the text of the handwritten records (as well as any text in the ‘Comments’ box of the AIMS records). In the event of a difference of opinions regarding whether an element was documented or not then a third reviewer (DN) adjudicated to make a final decision. Each record was then assigned a total score out of 15 (which we termed the documentation completeness score, DCS).

**Table 1.** 15 epidural insertion documentation components selected for analysis in both handwritten and AIMS records.

​	Documentation Components	Handwritten Record Examples	AIMS Interface Elements
1.	Patient position	‘Patient sitting’, ‘Lateral position’	Dropdown (Sitting, Lateral)
2.	Aseptic technique observed	‘Aseptic technique’, ‘Sterile gown/gloves’ etc	Checkbox
3.	LA skin infiltration	‘1% lidocaine to skin’, ‘LA to skin’	Checkbox
4.	Approach	‘Midline’, ‘Paramedian approach’	Dropdown (Midline, Paramedian)
5.	Spinal level	‘T5/6’, ‘L2-3’ etc	Dropdown (T5/6, L1/2 etc.)
6.	Needle type	‘Tuohy’	Dropdown (Tuohy)
7.	Needle size	‘18G’	Dropdown (16G or 18G)
8.	Loss of resistance technique	‘LOR to air’, ‘Loss of resistance to saline’	Dropdown (Saline, Air)
9.	Loss of resistance depth	‘LOR at 5cm’, ‘LOR 6cm’	Numeric (cm)
10.	Catheter depth at skin	‘Catheter left at 10cm at skin’	Numeric (cm)
11.	Fluid return via needle/catheter	‘No blood or CSF’, ‘No blood or CSF via needle’	Dropdown (None, blood, CSF)
12.	Paresthesia	‘Transient paresthesia with catheter’	Dropdown (None, with needle, with catheter)
13.	LA test dose	‘3ml 0.25% bupivacaine as test dose’	Documented in main anesthesia record
14.	Response to LA test dose	‘No response to test dose’	No specific element (Comments box provided)
15.	LA treatment dose	‘8ml/hr 0.125% bupivacaine infusion’	Documented in main anesthesia record

Examples of how these elements are typically documented on paper are shown in the middle column. The right-hand column shows the options available on the ‘Central blocks’ tab in AIMS for documenting these items. LA, local anaesthetic; CSF, cerebrospinal fluid; AIMS, anaesthesia information management system; LOR, loss of resistance.

Of note, there are two mandatory dropdown elements on the ‘Central blocks’ tab: the ‘Select procedure’ dropdown prompts the user to select either spinal or epidural anaesthesia, and ‘Needle details’ prompts the user to select needle type, either epidural or spinal needle. Selecting epidural needle enables the ’Needle type’ and ‘Needle size’ elements which are pre-populated with appropriate options (‘Tuohy’, and ‘16G’ or ‘18G’ respectively). Neither of these two mandatory dropdowns (‘Select procedure’ and ‘Needle details’) were used to calculate the DCS.

### Sample Size Estimation and Statistical Analysis

A previous study comparing handwritten anesthetic records to an AIMS in terms of overall compliance with completion of a checklist of 35 elements covering a wide variety of information captured by anesthesia records detected a mean difference of approximately 7% between the two (AIMS being superior, 88.6% versus 81.5%).^16^ Assuming a similar pattern of completion, 63 records would be required in each group in order to provide a power of 80% to detect a DCS difference of 7% between handwritten and AIMS records. To provide a reasonable margin of error we rounded this up to 100 records per group.

Categorical variables were summarized as percentages and continuous variables were described as mean and standard deviation (SD) when the data followed a normal distribution, otherwise by using median and interquartile range (IQR). The distribution of values in continuous variables was evaluated using the Kolmogorov–Smirnov test. Two-tailed Fisher’s exact test was used to compare categorical data, and confidence intervals of relative risks were calculated using Koopman asymptotic scoring. For continuous non-normally distributed variables (such as DCS), the Mann–Whitney U test was used. P-values of <0.05 were considered statistically significant. When multiple comparisons were performed (as with the analysis of the 15 individual documentation components), the statistical significance level was adjusted using Bonferroni correction. Data was analysed using Prism 10.4.1 (GraphPad, California, USA).

## Results

Of 100 handwritten records retrieved, 97 cases were included in final analysis. 3 cases were excluded: one described a caudal epidural (rather than a standard thoraco-lumbar technique), one was partially illegible, and one was abandoned early in favor of another technique. Word counts used to describe epidural placement varied widely, range 5 to 43 (median 24, IQR 19 to 31). Each of the 97 handwritten records was completed by one of 27 consultant anesthesiologists working in conjunction with one of 53 resident anesthesiologists (this center is a major university teaching hospital where residents rotate through for 6- or 12-month training blocks). Of the 100 AIMS records retrieved, all were deemed suitable for inclusion in the final analysis. Each AIMS record was completed by one of 23 consultant anesthesiologists working with one of 47 resident anesthesiologists. In both handwritten and AIMS records it was not possible to determine which of the consultant or resident present completed the epidural documentation. The ‘Comments’ box was used to provide additional text in 44/100 (44%) AIMS records. The results of the comparison of each of the 15 documentation components between handwritten and AIMS records are shown in Figure 2 and Table 2. In 11 out of 15 components, AIMS significantly outperformed handwritten records. Handwritten records outperformed AIMS with respect to one component (‘LA test dose documented’). Three components were not significantly different between paper and AIMs (‘Catheter depth at skin’, ‘LA treatment dose documented’ and ‘Response to LA test dose’). Analysis of DCS showed that AIMS records had significantly higher scores than handwritten records, mean 13.48 (SD 1.01) versus 9.96 (2.95), p<0.001 (see Figure 3).

**Figure 2.**
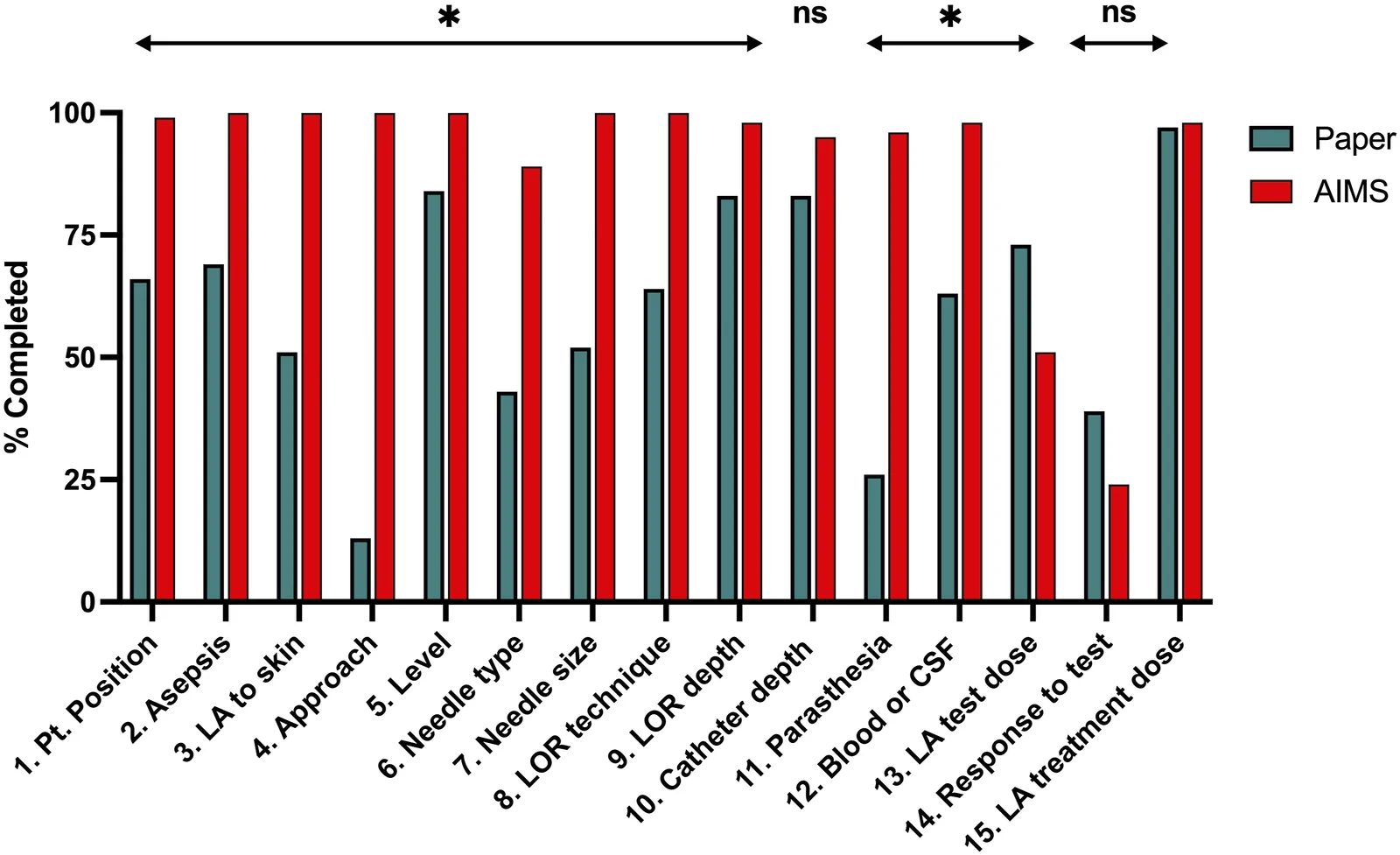
Graph of handwritten versus AIMS record documentation results by individual component. * indicates statistical significance between handwritten and AIMS component. (p<0.0033, after Bonferroni correction). **ns** indicates non-significance. AIMS, anaesthesia information management system; LA, local anaesthetic; LOR, loss of resistance; CSF, cerebrospinal fluid.

**Table 2.** Handwritten versus AIMS record documentation results by individual component.

​	Documentation Component	AIMS (n=100)	Handwritten (n=97)	p-value	Relative Risk (95% CI)
1.	Patient position	99 (99.0%)	66 (68.0%)	**<0.0001**	1.46 (1.29 to 1.70)
2.	Asepsis observed	100 (100%)	69 (71.1%)	**<0.0001**	1.41 (1.41 to 1.50)
3.	LA to skin	100 (100%)	51 (52.6%)	**<0.0001**	1.90 (1.73 to 1.95)
4.	Approach	100 (100%)	13 (13.4%)	**<0.0001**	7.46 (4.84 to 12.3)
5.	Level	100 (100%)	84 (86.6%)	**<0.0001**	1.15 (1.15 to 1.15)
6.	Needle type	89 (89.0%)	43 (47.8%)	**<0.0001**	2.01 (1.62 to 2.57)
7.	Needle size	100 (100%)	52 (57.8%)	**<0.0001**	1.87 (1.86 to 2.08)
8.	LOR technique	100 (100%)	64 (66.0%)	**<0.0001**	1.52 (1.51 to 1.52)
9.	LOR depth	98 (98.0%)	83 (85.6%)	**0.0014**	1.15 (1.06 to 1.27)
10.	Catheter depth at skin	95 (95.0%)	83 (85.6%)	0.0301	1.11 (1.01 to 1.24)
11.	Parasthesia	96 (96.0%)	26 (26.8%)	**<0.0001**	3.58 (2.63 to 5.06)
12.	Blood or CSF via needle/catheter	98 (98.0%)	63 (64.9%)	**<0.0001**	1.51 (1.32 to 1.78)
13.	LA test dose documented	51 (51.0%)	73 (75.3%)	0.0006*	0.68 (0.54 to 0.84)
14.	Response to LA test dose	24 (24.0%)	39 (40.2%)	0.0215	0.59 (0.39 to 0.91)
15.	LA treatment dose documented	98 (98.0%)	97 (100.0%)	0.4976	0.98 (0.93 to 1.02)

Stated p-value is calculated using two-tailed Fisher’s exact test. P-values in **bold script** indicate statistical significance following Bonferroni correction (p<0.0033). * indicates components where handwritten records outperform AIMS; otherwise, AIMS outperforms handwritten records where p<0.0033. AIMS, anaesthesia information management system; CI, confidence interval; LA, local anaesthetic; LOR, loss of resistance; CSF, cerebrospinal fluid.

**Figure 3.**
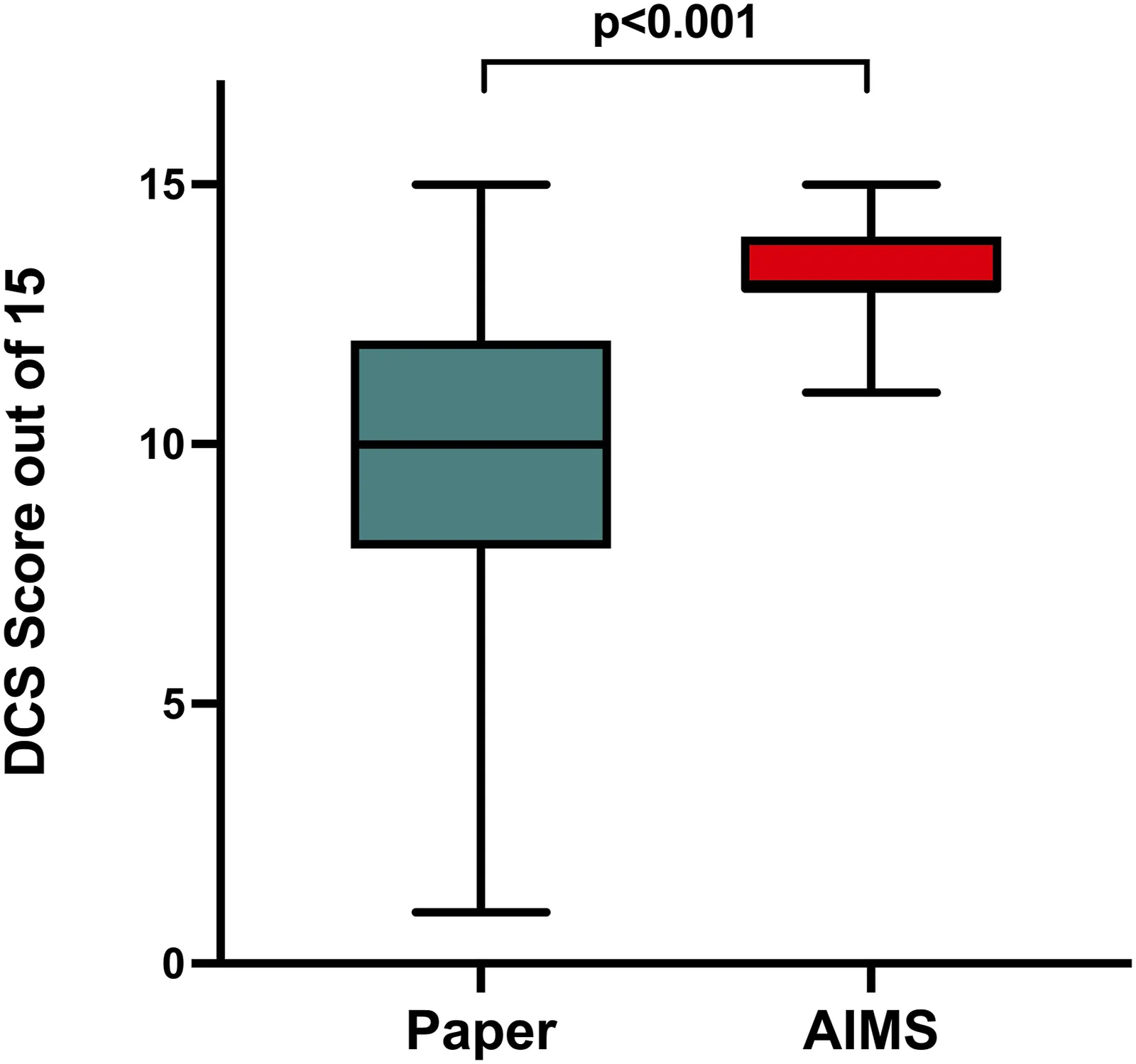
Box-whisker plot of Documentation Completion Scores (DCS) by group. p<0.001 by Mann-Whitney U-test.

## Discussion

Our results indicate that overall an AIMS record more consistently captures epidural documentation elements compared to handwritten records. Completion rates for 12 out of 15 elements were equal to or greater than 95% with AIMS, compared to only 1 with handwritten records (‘LA treatment dose documented’). This was despite the fact that selection of these elements was not mandatory in the AIMS interface page (and therefore the user could choose to ignore them and leave them uncompleted if they wished). It is evident that most components integrated into the AIMS interface can easily and quickly be entered using drop-down boxes or numeric entry fields. Having a set of pre-defined elements displayed (rather than a free-text box to describe the procedure) acts as an aide-memoire and appears to reduce the risk of the user forgetting to include elements which they would otherwise deem important (a so-called ‘nudge’ in behavioral economics terms).^17^

Certain elements were particularly poorly documented in handwritten records: approach (13/97, 13.4%), paresthesia (26/97, 26.8%) and response to test dose (39/97, 40.2%). The failure to document approach may be explained by the fact that the very large majority of epidurals in our institution are placed using a midline approach (>95% from a previous audit of practice). Thus, users may have considered that specifically documenting the approach is unnecessary when midline is the institutional convention. However, the failure to document paresthesia and response to test dose is surprising, given the relative importance that would be attributed to these elements if a complication was to occur and a clinical or medicolegal review was performed. Failure to document a simple description such as ‘no paresthesia, no motor block 5 mins after test dose’ would likely weaken the ability of the practitioner to defend their management in the event of a complaint.

Perhaps even more surprising is that AIMS performed significantly worse than handwritten records in terms of ‘LA test dose documented’ (51.0% vs 75.3%, p<0.0006). AIMS may at first inspection have also underperformed in terms of ‘Response to test dose’ (24.0% vs 40.2% completion); however, this did not reach statistical significance after correction for multiple comparisons. Why handwritten records also performed poorly (40.2% completion) is unclear, and it is impossible to determine if this reflects a failure to document the response, or a failure to check for the response in the first place. The AIMS configuration expects the user to document drug administration in the ‘Medications’ section of the main AIMS chart and does not provide this functionality within the ‘Central blocks’ tab. Also note that there is no specific interface element corresponding to ‘Response to test dose’, although a ‘Comments’ box is provided to allow users to document what they wish (the 24.0% of AIMS users who documented response to test dose did so in the Comments box in a manner such as ‘2ml 0.5% bupivacaine given as test dose, no response’). It may be that because the user is not prompted to document test dose (or the response) by specific interface elements in the existing ‘Central block’ tab that they forget to do so elsewhere. However, this does not explain why the ‘treatment dose’ is so well documented in AIMS (98%) given that there is no specific interface element provided for this either. Nonetheless, it seems reasonable to expect that reconfiguring the AIMS interface (adding a dedicated option for ‘Response to test dose’, and a reminder to document LA doses in the main chart) would improve the completion of these underperforming elements. Reminders have been shown to be a powerful means of improving documentation quality,^18,19^ however, the AIMS version in this study was not configured to provide an easy-to-use reminder system, and it was not directly integrated within a larger electronic health record (EHR) system, which may have provided similar reminder functionality. Such functionality may become available in future versions. When the AIMS was initially configured for local use, consideration was given to making far more documentation elements mandatory in order to enforce documentation quality. However, this approach was deliberately not taken as it was felt that forcing users to remain on certain tabs until data was entered would disrupt the early user experience, cause frustration, and erode positive sentiment towards adoption of the AIMS system, especially with ongoing rotation of resident doctor users from other centers where handwritten records remain in use. In the future, and as electronic health care records in general become more embedded in our center and others, it may be that organizational and operational priorities will shift away from a focus on optimizing user experience and system adoption, and move towards enforcing documentation completion and quality.

A number of limitations should be noted when considering the results of this study. Firstly, we assume that the only variable causing a difference between the two groups is the introduction of AIMS. While AIMS certainly appears to be the greatest factor affecting documentation completion in this study, we cannot establish causality nor exclude the possibility that unidentified confounders may also be involved. For instance, it is possible that anesthesia resident training changed between the two time periods to create a greater focus on documentation quality. Indeed, new users are provided with a number of instructional videos which detail how to use the AIMS system in general (such as how to find and start cases, connect monitors, add drugs etc). Although these do not address the documentation of epidurals specifically, it may be that even a general discussion of anaesthesia documentation prior to commencing clinical work enhances the user’s awareness and appreciation of documentation quality. Secondly, although random selection was used, as with many retrospective studies there remains a risk that selection bias may still be present resulting in non-representative samples. Thirdly, it may well be that if the original paper record document provided a granular template for recording epidural placement, there would have been little difference noted in DCS with the introduction of AIMS. However, our original generic paper record document was designed to be used across a wide variety of anaesthetic and procedure types, and the addition of an epidural template would take up limited document space which would only be used in the minority of cases where epidurals were placed and would otherwise go unused. Fourthly, given the considerations outlined above, the generalizability of the results to other institutions is unclear. Another center using a detailed paper epidural template switching to a poorly configured AIMS may well find a decrease in documentation quality. Finally, each of the 15 documentation elements is given equal weight in terms of calculating DCS, whereas it could be argued that some carry more significance than others, e.g. documenting the absence or presence of paresthesia during the procedure is likely more significant medico-legally than documenting whether skin was infiltrated with local anesthetic. Given that assigning weights would be an arbitrary and controversial exercise, we decided to adopt a more neutral approach, treating each element as equal.

Notwithstanding the potential limitations, our study addresses two recent trends within the field of anesthesia, which are likely to be converging and also long overdue. The first is the inexorable move to universal use of electronic health records (including AIMS), although without effective national oversight in many countries this remains a fragmented effort and it is expected that a considerable number of organizations will be reliant on paper charts for the near-to medium-term future.^20^ The second is the recent development of consensus-based documentation guidelines from expert groups and national bodies which should assist in the configuration of existing and new recording systems to capture vital procedural data better.^13,14,21^ Together, these two trends will not only help to enhance patient safety and the delivery of high-quality patient care, but also provide invaluable practice aids for the anesthesiologist. Future exciting advances in anaesthesia recording and documentation beyond these are already taking shape, as novel applications of machine vision, machine learning, and artificial intelligence begin to be applied in the operating room environment.^22,23^

## Conclusion

Applied within our institution, epidural documentation using a pre-configured AIMS application provides an objectively more comprehensive procedural record compared to handwritten documentation when assessed using a novel completion score. However, the configuration of AIMS may unintentionally lead users to fail to document epidural insertion elements if all epidural-related documentation in not contained within a single page of the AIMS interface. The more widespread adoption of AIMS, together with standards-based anesthesia documentation guidance, should improve the overall quality of anesthesia documentation to the benefit of patients and practitioners alike.

## Supplemental material

Supplemental material - The effect of Anesthesia Information Management Systems (AIMS) on perioperative epidural documentation: A retrospective cohort study Supplemental material for The effect of Anesthesia Information Management Systems (AIMS) on perioperative epidural documentation: A retrospective cohort study by Dulmi Nawarathna, John Shaker, and Thomas P. Wall in DIGITAL HEALTH.

## Data Availability

Anonymised, aggregate data will be made available by the corresponding authors upon reasonable request.*
